# Supplementary material for: Tumour-vasculature development via endothelial-to-mesenchymal transition after radiotherapy controls CD44v6+ cancer cell and macrophage polarization
Source: Nat Commun. 2018 Nov 30;9:5108. doi: 10.1038/s41467-018-07470-w (PMC6269447; doi:10.1038/s41467-018-07470-w)
Supplement: Supplementary file 1 — Supplementary Information [file 41467_2018_7470_MOESM1_ESM.pdf]

Supplementary information

Tumour-vasculature development via endothelial-to-mesenchymal transition after radiotherapy controls CD44v6^{+} cancer cell and macrophage polarization

Choi *et al.*

Supplementary Figure 1

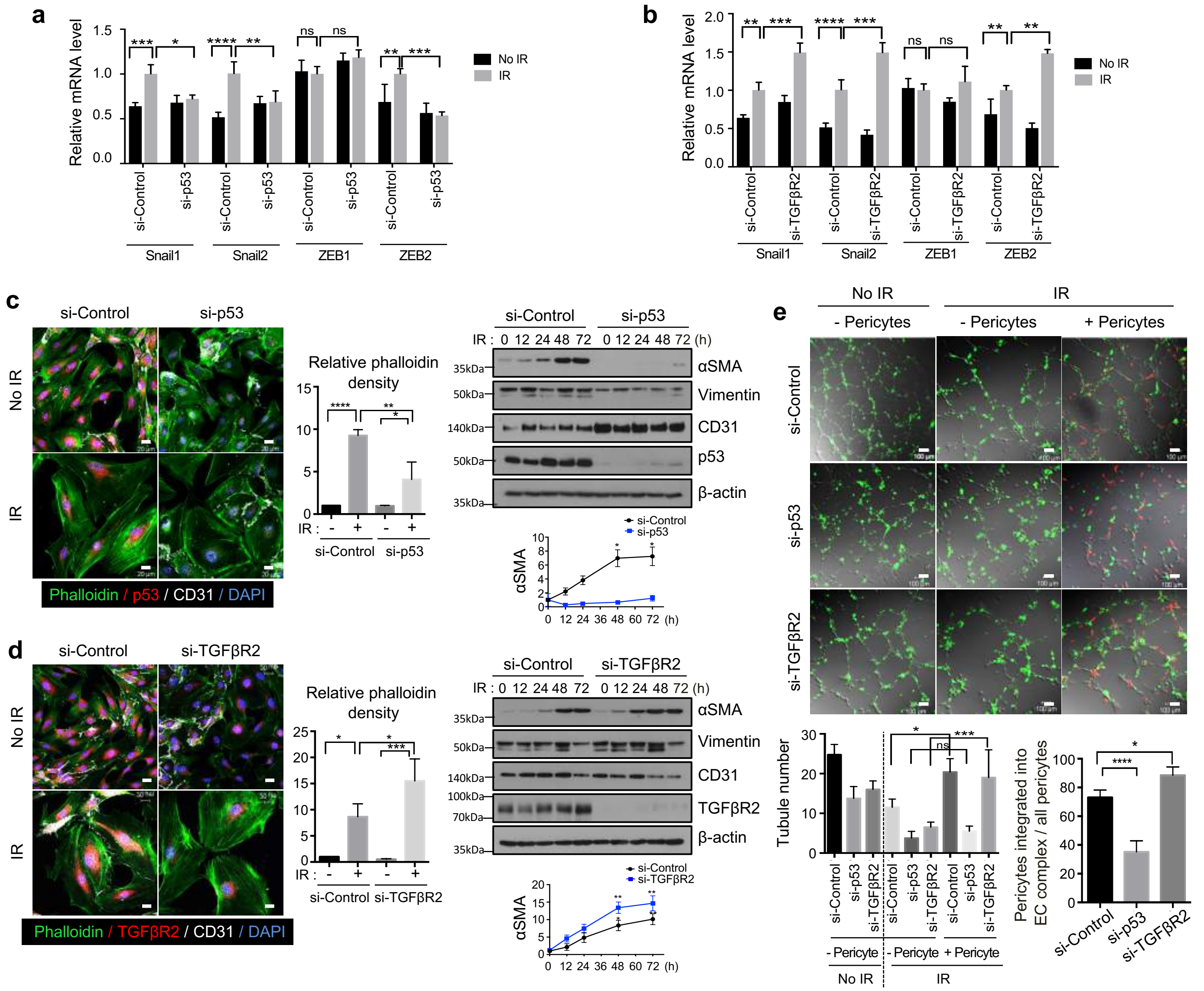

Supplementary Figure 1. Radiation-induced EndMT regulates pericyte integration into EC tubules and *in-vitro* transendothelial migration of tumour cells. (**a, b**) RT-qPCR analysis of *Snail1*, *Snail2*, *ZEB1*, and *ZEB2*, 48 h after 10-Gy irradiation in HUVECs by *Trp53* knockdown (KD) (**a**) and *Tgfbr2* KD (**b**). (**c**) Immunofluorescence detection of phalloidin, p53, and CD31 in 10-Gy-irradiated HUVEC-p53KD cells (day 3; left) and quantification of the phalloidin intensity per field (magnification, 200×, center; error bars indicate the mean ± SEM, n = 5). Immunoblotting of αSMA, vimentin, CD31, p53, and β-actin in HUVEC-p53KD cells at the indicated times (hpi) (10 Gy; right). Mean intensity of αSMA from three independent experiments is given (bottom). Scale bar = 20 μm. (**d**) Immunofluorescence detection of phalloidin, TGFβR2, and CD31 in 10-Gy-irradiated HUVEC-TGFβR2KD cells (day 3; left) and quantification of the phalloidin intensity per field (magnification, 200×, center; error bars indicate the mean ± SEM, n = 5). Immunoblotting of αSMA, vimentin, CD31, p53, and β-actin in HUVEC-TGFβR2KD at the indicated times (hpi) (10 Gy; right). The mean intensity of αSMA from three independent experiments is shown (bottom). Error bars indicate the mean ± SD. Scale bar = 20 μm. (**e**) Tube formation in *Trp53* or *Tgfbr2* KD ECs, in the presence or absence of pericytes, after 10 Gy irradiation (upper panels), and quantification of the number of tubes per field and fraction of pericytes integrated into EC complex (magnification, 200×). Scale bar = 100 μm. Error bars indicate the mean ± SEM, n = 5. For all graphs, *p < 0.05, **p < 0.01, ***p < 0.001, ****p < 0.0001, ns = not significant (one-way ANOVA for multiple comparision). All data are representative of three independent experiments.

Supplementary Figure 2

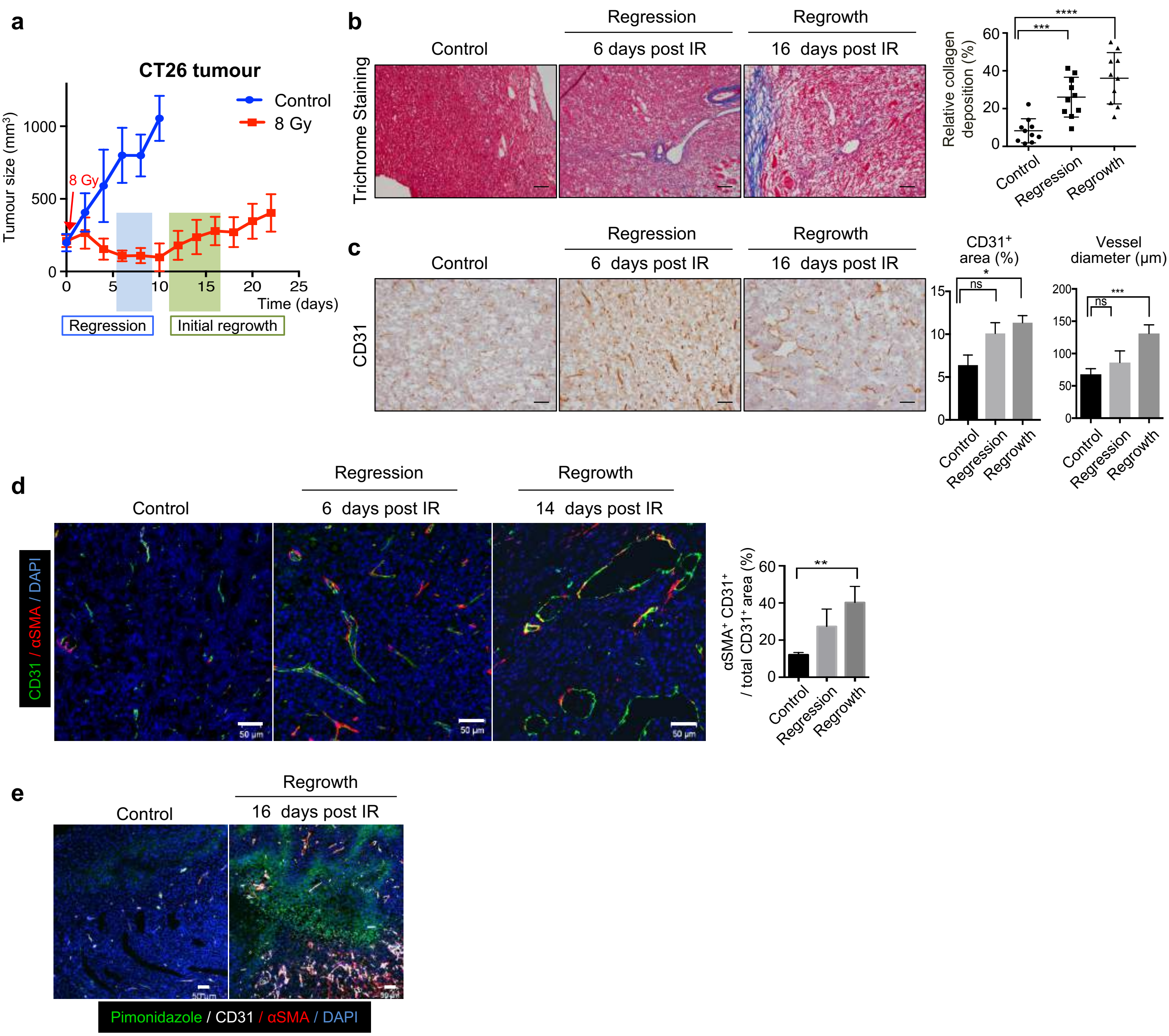

Supplementary Figure 2. EndMT, collagen deposition, and increased abnormal vasculature during CT26 tumour regrowth following a single 8 Gy irradiation. (**a**–**e**) CT26 cells were subcutaneously injected into the right hind legs of BALB/c mice. Tumour tissues were obtained at the indicated days after irradiation (n = 6–7 per time point). (**a**) CT26 tumour regression (blue region) and initial regrowth (green region) after irradiation (n = 10 per group). (**b**,**c**) Representative images of trichrome staining (**b**) and immunohistochemical detection of CD31 (**c**) in control, regressed, and regrown CT26 tumours at the indicated times. Quantitative data on collagen deposition, the $CD31^+$ area per 100× field, and vessel diameters (from five large vessels in one 100× field) are shown. Scale bars, 100 μm. Error bars for trichrome staining data indicate the mean ± SD. In all fields, n = 10. (**d**,**e**) Immunofluorescence detection of pimonidazole, CD31, and αSMA at the indicated times, with αSMA^+CD31^+ cell numbers calculated per $CD31^+$ cell in each field (magnification, 100×; n = 7). IR, irradiated (8 Gy). Scale bar = 50 μm. For all graphs, *$p < 0.05$, **$p < 0.01$, ***$p < 0.001$, ****$p < 0.0001$, ns = not significant (one-way ANOVA for multiple comparision). Unless indicated otherwise, error bars indicate the mean ± SEM. All data are representative of three independent experiments.

Supplementary Figure 3

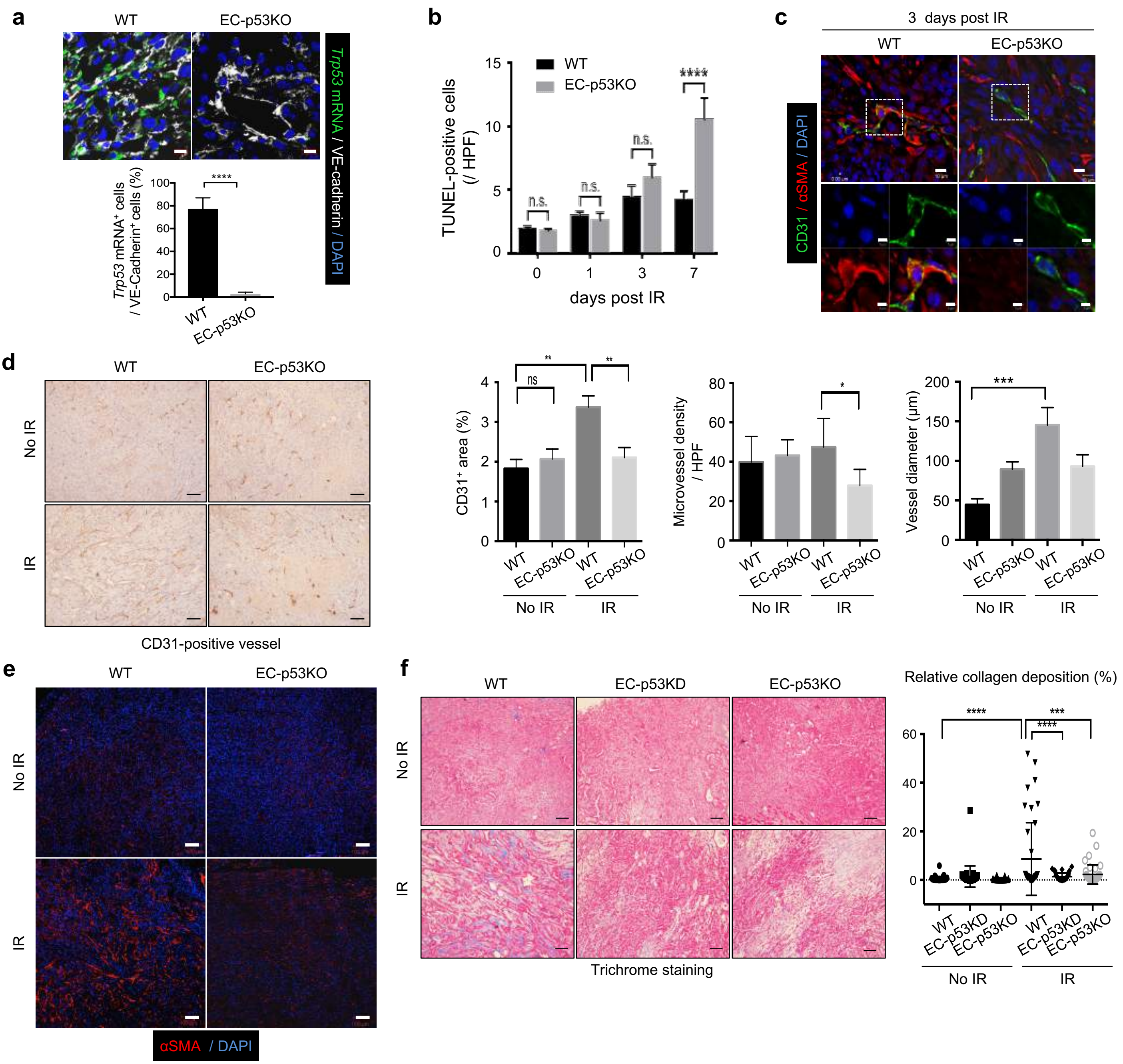

Supplementary Figure 3. Post-irradiation CD31 and αSMA distributions in KP tumours of EC-p53KO mice are different from those in KP tumours of WT mice. (**a**) RNAscope *in-situ* hybridization for *Trp53* mRNA and immunofluorescence detection for VE-cadherin in KP tumours, with quantification of the *Trp53* mRNA$^+$ cells per VE-cadherin$^+$ cells (magnification, 200×; n ≥ 5). Scale bar = 10 μm. (**b**) Quantification of TUNEL$^+$ cells per field (magnification, 200×) using immunofluorescence images of KP tumours from WT and EC-p53KO mice, with or without irradiation (1, 3, and 7 days post IR, dpi). (**c**) Immunofluorescence visualization of CD31 and αSMA in KP tumours from WT and *Tie2*-Cre;*Trp53$^{flox/flox}$* (EC-p53KO) mice 3 dpi. Scale bar = 10 μm (crop, 5 μm). (**d**) Immunohistochemical analysis of CD31 expression in tumours from WT and EC-p53KO mice, with quantification of the CD31$^+$ area (left panel), microvessel density per field (magnification, 100×) (middle panel), and vessel diameters (right panel). For right panel, the diameters of five large vessels per 100× field were measured. Scale bar, 100 μm. (**e**) Immunofluorescence visualization of αSMA in KP tumours from WT and transgenic mice after irradiation. Scale bar = 100 μm. (**f**) Trichrome staining of KP tumours on day 23 after irradiation and day 15 without irradiation. Scale bars, 100 μm. Collagen deposition shown as an average per five fields (magnification, 100×) from **f**. Error bars indicate the mean ± SD, n = 5 per group. For all graphs, *p < 0.05, **p < 0.01,. ***p < 0.001, ****p < 0.0001, ns = not significant (one-way ANOVA for multiple comparision). Unless otherwise indicated, error bars indicate the mean ± SEM. IR, irradiated. The mice were treated with 20 Gy irradiation given in a single fraction. All data are representative of three independent experiments.

Supplementary Figure 4

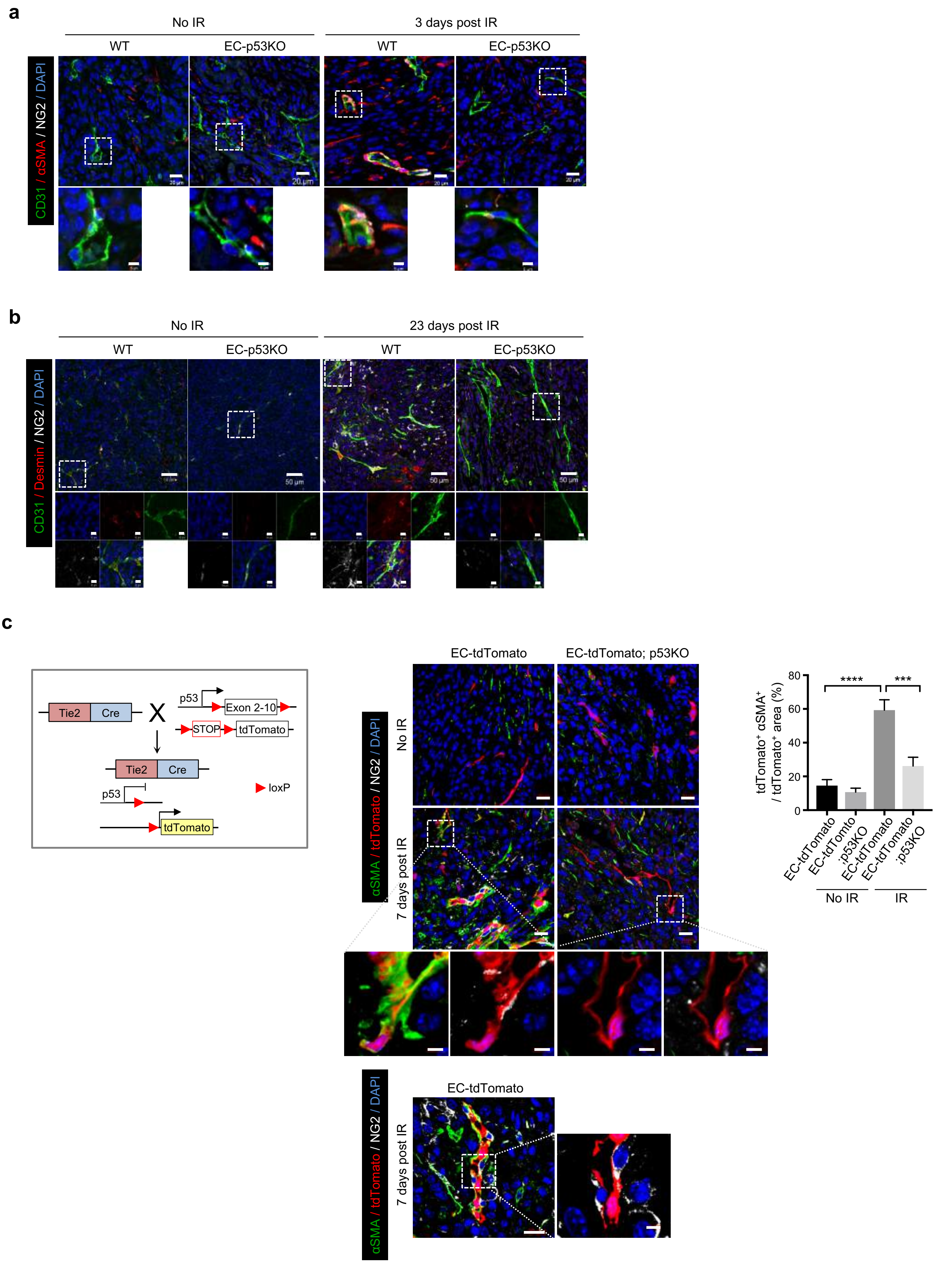

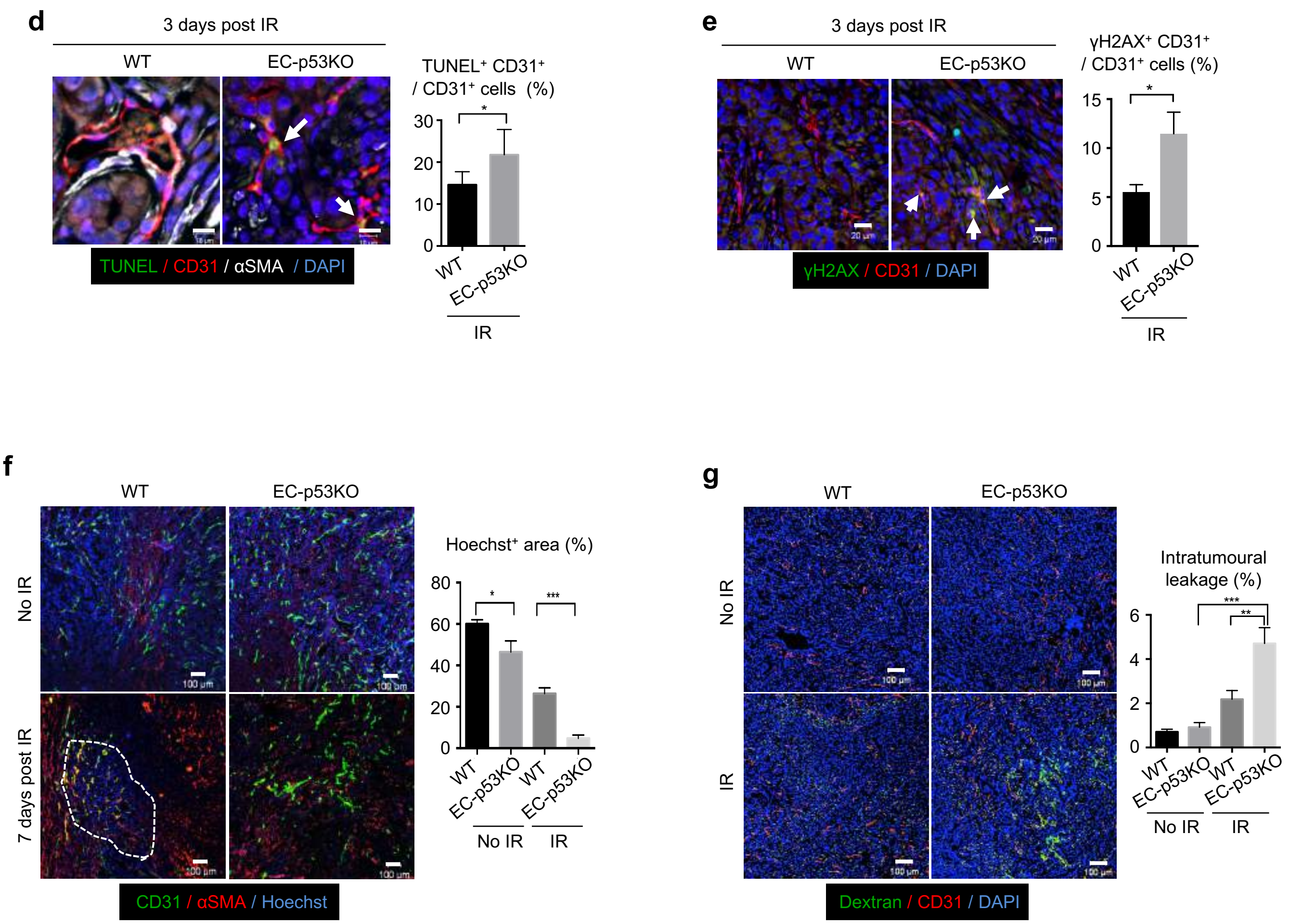

Supplementary Figure 4. EndMT in KP tumours of EC-p53KO mice increases after irradiation. (**a**) Immunofluorescence visualization of CD31, αSMA, and NG2 expression in KP tumours from WT and EC-p53KO mice 3 dpi. Scale bar = 20 μm (crop, 5 μm). (**b**) Immunofluorescence visualization of CD31, desmin, and NG2 in KP tumours from WT and EC-p53KO mice 23 dpi. Scale bar = 50 μm (crop, 10 μm). (**c**) Genetic strategy to delete *Trp53* in tdTomato-expressing ECs (left panel) and immunofluorescence visualization of tdTomato, αSMA, and NG2 expression in KP tumours from *Tie2*-Cre;R26R^{tdTomato} (EC-tdTomato) and *Tie2*-Cre;R26R^{tdTomato};*Trp53*$^{flox/flox}$ (EC-tdTomato; p53KO) mice 7 dpi (middle panel). Quantification of the tdTomato^{+} αSMA^{+} area per tdTomato^{+} field is shown (magnification, 200×; n ≥ 5) (right panel). Scale bar = 20 μm (crop, 5 μm). (**d**) Immunofluorescence detection of TUNEL, CD31, and αSMA in KP tumours from WT and EC-p53KO mice 3 dpi, with quantification of the TUNEL^{+} CD31^{+} area per CD31^{+} field (magnification, 200×; n ≥ 5). Scale bar = 10 μm. (**e**) Immunofluorescence visualization of CD31 and γH2AX in KP tumours from WT and EC-p53KO mice 3 dpi. Scale bar = 20 μm. (**f**) Immunofluorescence detection of CD31 and αSMA in KP tumours from WT and EC-p53KO mice 7 dpi. Mice were intravenously injected with perfusion marker Hoechst 33342. Quantification of the Hoechst 33342-perfused area per field is shown (magnification, 100×; n ≥ 5). Scale bar = 100 μm. (**g**) Immunofluorescence detection of CD31 in KP tumours from WT and EC-p53KO mice 7 dpi. Mice were intravenously injected with intratumoural leakage marker dextran. Quantification of the dextran-perfused area per field is shown (magnification, 100×; n ≥ 5). Scale bar = 100 μm. Images are representative of three independent experiments. IR, irradiated. Tumours were treated with 20 Gy irradiation given in a single fraction. For all graphs, *$p < 0.05$, **$p < 0.01$, ***$p < 0.001$, ****$p < 0.0001$ (one-way ANOVA for multiple comparision). Error bars indicate the mean ± SEM. All data are representative of three independent experiments.

Supplementary Figure 5

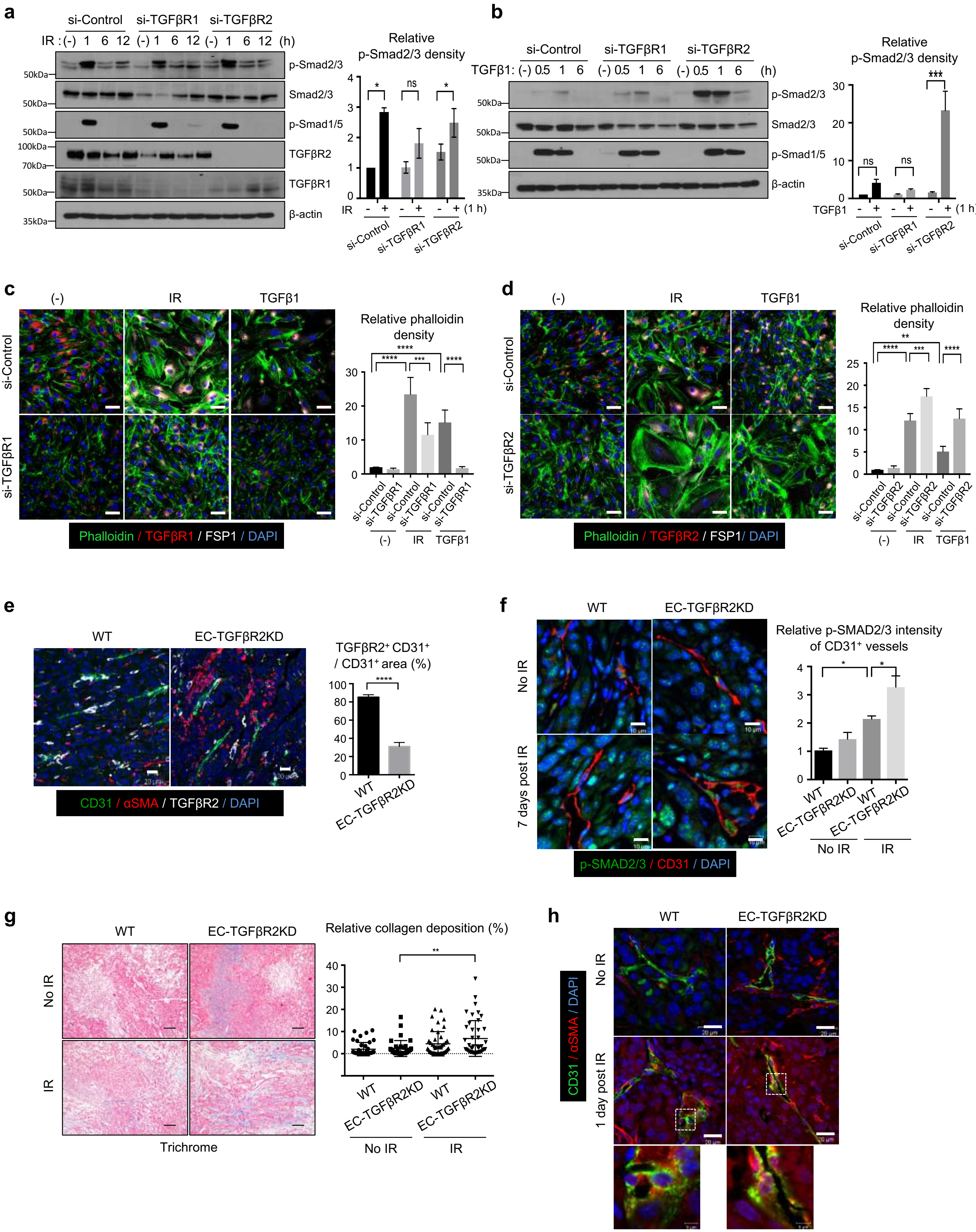

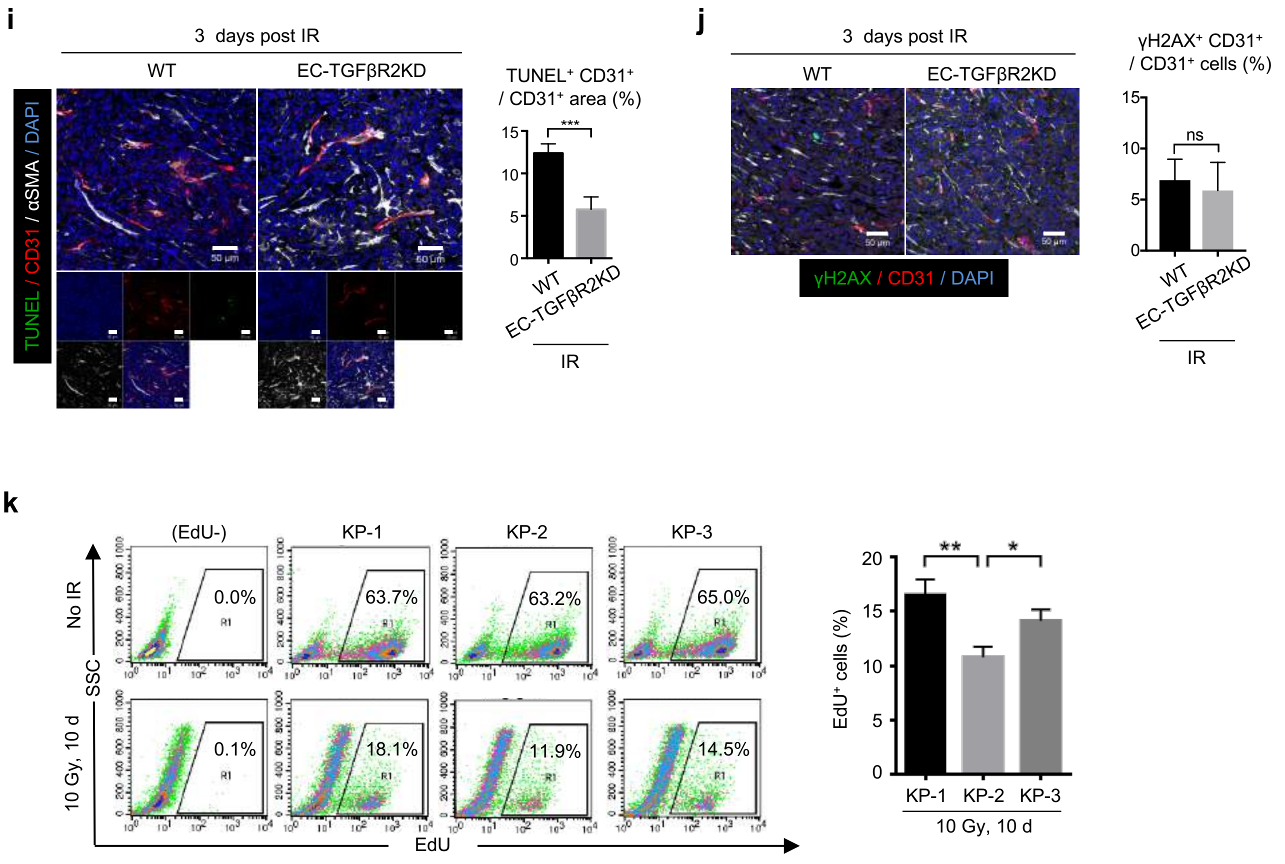

Supplementary Figure 5. Increased EndMT, collagen deposition, and TGFβ1-SMAD2/3 activities in irradiated KP tumours of EC-TGFβR2KD mice. (**a**,**b**) Immunoblotting of p-SMAD2/3, SMAD2/3, p-SMAD1/5, TGFβR1, TGFβR2, and β-actin in HUVEC-TGFβR1 or 2 KD cells at the indicated times (hpi) (10 Gy) (**a**) or post-TGF-β1 treatment. The mean intensity of p-SMAD2/3 from 3 independent experiments is shown (right) (**b**). (**c**) Immunofluorescence visualization of phalloidin, TGFβR1, and FSP1 in HUVEC-TGFβR1 KD cells at 3 dpi (10 Gy) or post-TGF-β1 treatment. Quantification of relative phalloidin densities per field is shown (magnification, 200 ×; n ≥ 5). Scale bar = 50 μm. (**d**) Immunofluorescence visualization of phalloidin, TGFβR2, and FSP1 in HUVEC-TGFβR2 KD cells at 3 dpi (10 Gy) or post-TGF-β1 treatment. Quantification of relative phalloidin densities per field is shown (magnification, 200×; n ≥ 5). Scale bar = 50 μm. (**e**) Immunofluorescence visualization of CD31, αSMA, and TGFβR2 in KP tumours derived from WT and *Tie2*-Cre;*Tgfbr*$^{flox/+}$ (EC-TGFβR2KD) mice. Quantification of TGFβR2^{+} CD31^{+} lesions per CD31^{+} area in each field (magnification, 100×). Scale bar = 20 μm. (**f**) Immunofluorescence visualization of phospho-(p)-SMAD2/3 and CD31 in 20 Gy irradiated KP tumours derived from WT and EC-TGFβR2KD mice. Quantification of relative p-SMAD2/3 densities of CD31^{+} vessels per field (magnification, 200×). Scale bar = 10 μm. (**g**) Trichrome staining of irradiated KP tumours derived from WT and EC-TGFβR2KD mice and quantification of collagen deposition from four fields (magnification, 100×). Error bars indicate the mean ± SD (WT mice, n = 4; WT mice + IR, n = 8; EC-TGFβR2KD mice, n = 4; EC-TGFβR2KD mice + IR, n = 8). (**h**) Immunofluorescence visualization of CD31 and αSMA in KP tumours derived from WT and EC-TGFβR2KD mice before and 1 dpi. Scale bar = 20 μm (crop, 5 μm). (**i, j**) Immunofluorescence visualization of TUNEL (**i**) staining with immunofluorescence detection of CD31 and αSMA, and γH2AX and CD31 (**j**) in KP tumours derived from WT and EC-TGFβR2KD mice, 3 dpi. Scale bar = 50 μm (crop, 10 μm). (**k**) Flow-cytometric analysis of EdU incorporation in primary KP tumour cells 10 days after 10 Gy irradiation. Three different KP tumour cells (KP-1, KP-2 and KP-3) were respectively used in the tumour growth experiments of Fig.1b, 2b and 2g. IR, irradiated. The mice were treated with 20 Gy irradiation given in a single fraction. For all graphs, *p < 0.05, **p < 0.01, ***p < 0.001, ****p < 0.0001, ns = not significant (one-way ANOVA for multiple comparision). Unless otherwise indicated, error bars indicate the mean ± SEM. All data are representative of three independent experiments.

Supplementary Figure 6

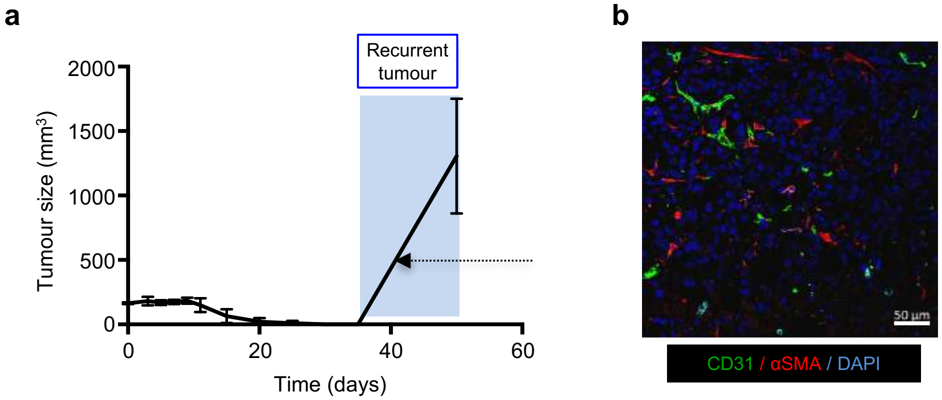

Supplementary Figure 6. Vasculature in recurrent tumours after radiation therapy. (**a**) KP cells were subcutaneously injected into the right hind legs of C57BL/6 mice (n = 10). Recurrent tumour tissues were obtained at 40 dpi (25 Gy, twice) (n = 5) (**b**) Immunofluorescence detection of CD31 and αSMA in recurrent tumour tissues at the indicated time points (marked with an arrow). Scale bar = 50 µm. IR, irradiation (25 Gy, twice). Error bars indicate the mean ± SD. All data are representative of two independent experiments.

Supplementary Figure 7

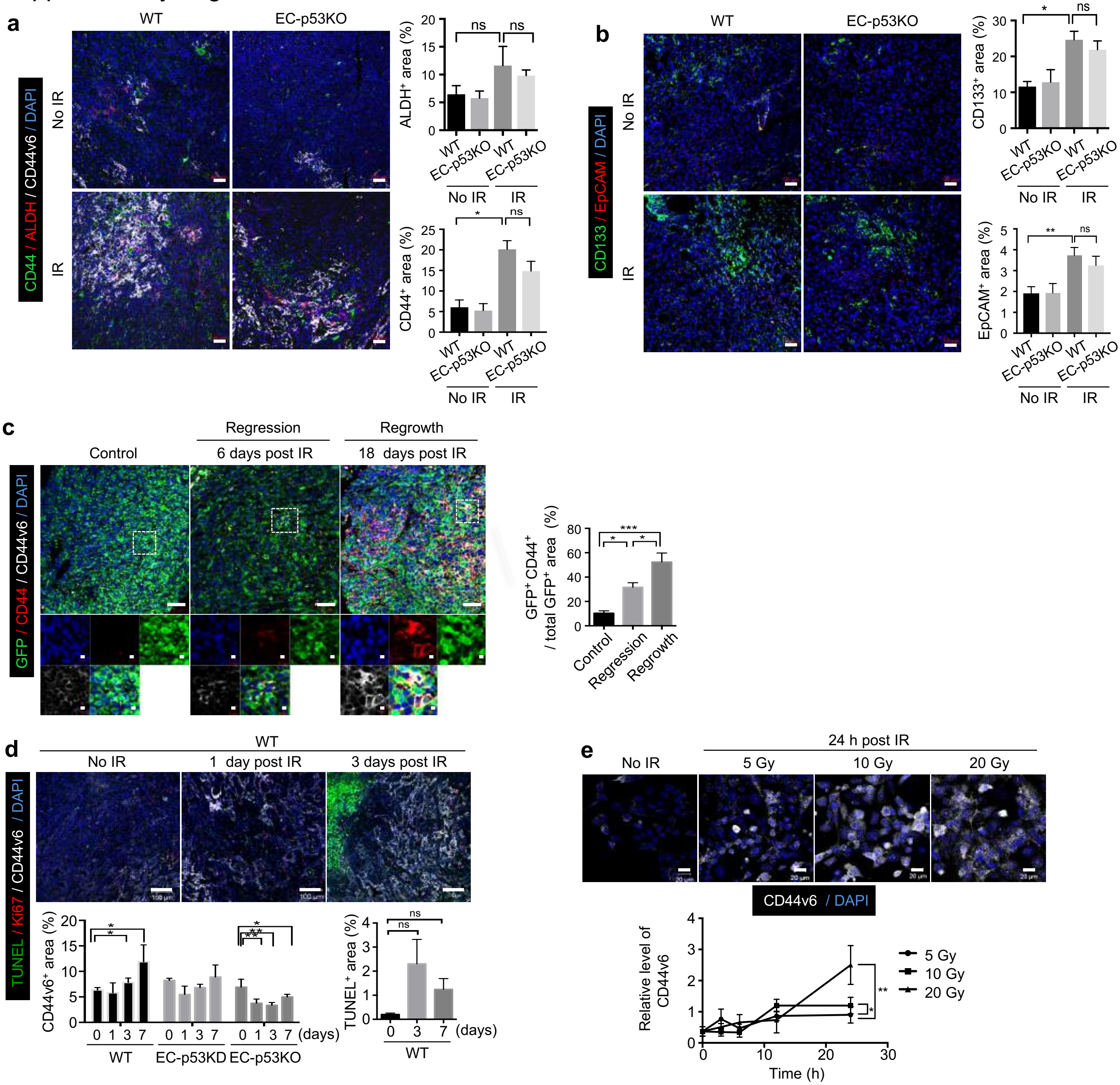

Supplementary Figure 7. Proliferating CD44v6⁺ tumours cells are increased after irradiation. (**a**) Immunofluorescence detection of CD44, aldehyde dehydrogenase (ALDH), and CD44v6 in KP tumours from WT and EC-p53KO mice 23 days after a single 20 Gy irradiation, with quantification of ALDH⁺ areas or CD44⁺ areas in each field (magnification, 200×; n > 5). Scale bar = 50 μm. (**b**) Immunofluorescence visualization of CD133 and EpCAM in KP tumours from WT and EC-p53KO mice, 23 days after 20 Gy irradiation with quantification of CD133⁺ areas or EpCAM⁺ areas in each field (magnification, 200×; n > 5). Scale bar = 50 μm. (**c**) Immunofluorescence visualization of GFP, CD44, and CD44v6 in GFP-stable CT26 tumours during regression (day 6 after 8 Gy irradiation) and regrowth (day 18 after 8 Gy irradiation). Quantitation of CD44⁺ cells per GFP⁺ area in each field (magnification, 200×; n ≥ 5). Scale bar = 50 μm (crop, 5 μm). (**d**) Immunofluorescence visualization of TUNEL, Ki67, and CD44v6 in KP tumours from WT mice 1, 3, and 7 days after 20 Gy irradiation. Quantitation of CD44v6⁺ cells per field in KP tumours sections from WT, EC-p53KD (*Trp53* knockdown mice), and EC-p53KO mice 0, 1, 3, and 7 dpi is shown (magnification, 200×; bottom). Scale bar = 100 μm. (**e**) Immunofluorescence visualization of CD44 and CD44v6 in KP tumour cells 3, 6, 12, and 24 hpi (5, 10, 20 Gy) and quantitation of CD44v6⁺ cells in KP tumour cells (lower graph). Scale bar = 20 μm. For all graphs, *p < 0.05, **p < 0.01, ***p < 0.001, ns = not significant (one-way ANOVA for multiple comparision). Unless indicated otherwise, error bars indicate the mean ± SEM. All data are representative of three independent experiments.

Supplementary Figure 8

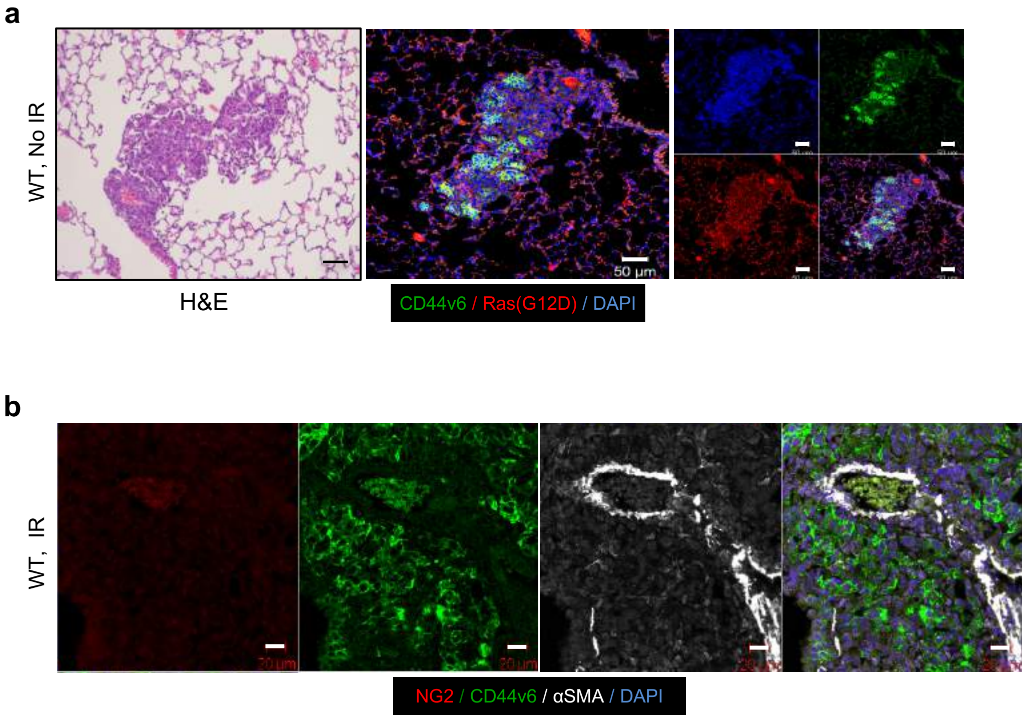

Supplementary Figure 8. Immunofluorescence of metastatic tumours of WT versus EC-TGFβR2KD mice. (**a**) Hematoxylin and eosin staining (left, scale bars, 100 µm) and immunofluorescence detection of CD44v6 and KRAS^{G12D} in metastatic lung KP tumours derived from WT mice (right). Scale bar = 50 µm. (**b**) Immunofluorescence detection of NG2, CD44v6, and αSMA in lung metastatic KP tumours from WT mice 23 days after 20 Gy irradiation given in a single fraction. Scale bar = 20 µm. All data are representative of three independent experiments.

Supplementary Figure 9

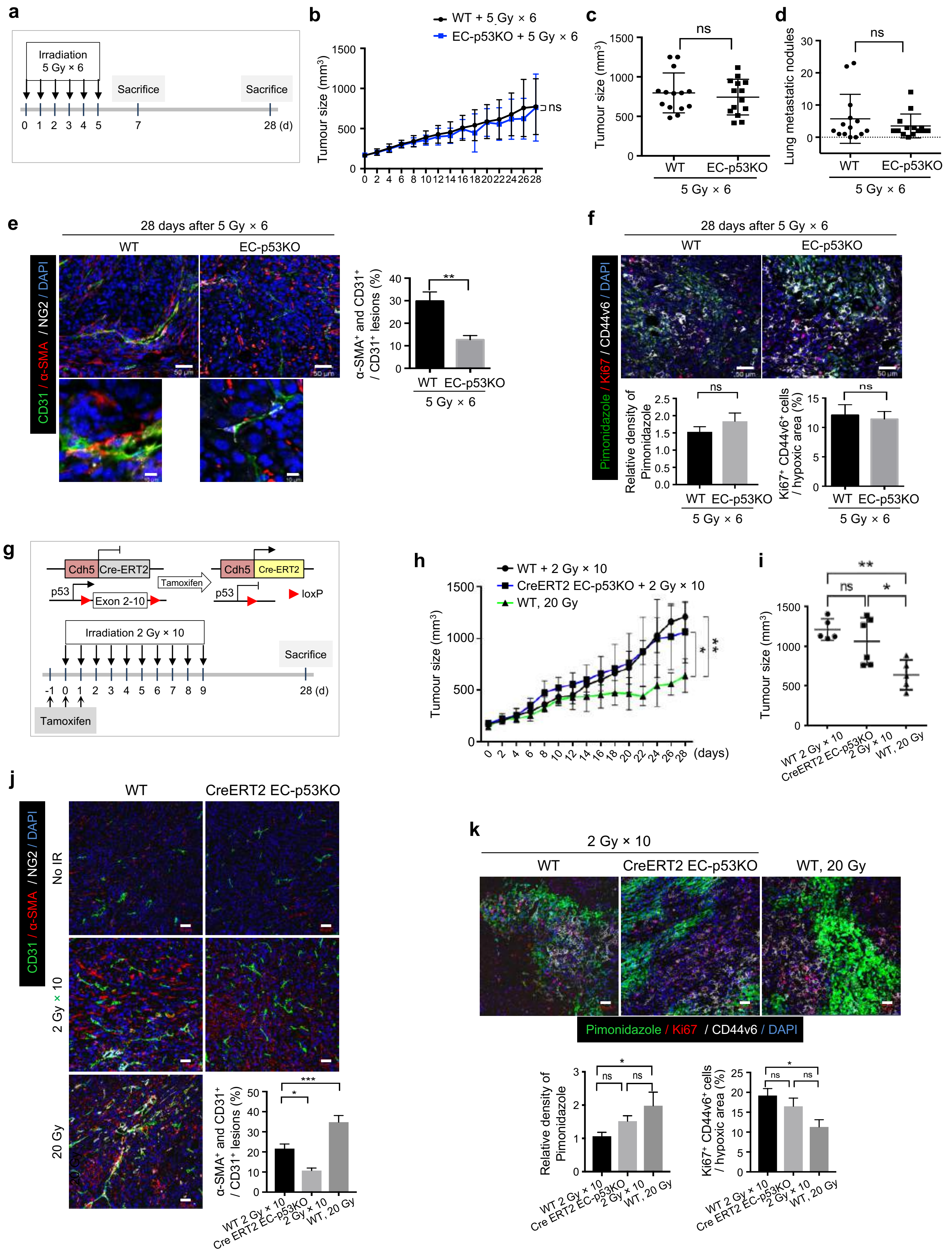

Supplementary Figure 9. Effects of fractionated-dose compared to single-dose irradiation on EndMT, hypoxia, and $CD44v6^{+}$ cancer cell proliferation. KP lung tumour cells were subcutaneously injected into the right hind legs of WT and *Tie2*-Cre;*Trp53*$^{flox/flox}$(EC-p53KO), and VE-cadherin-Cre-ERT2;*Trp53*$^{flox/flox}$ (CreERT2 EC-p53KO) mice. **(a)** WT and EC-p53KO mice received five fractions of 6 Gy on consecutive days. **(b)** Growth of KP tumours in WT and EC-p53KO mice after fractionated irradiation. **(c)** Tumour sizes on day 28 (after fractionated irradiation). **(d)** Lung metastatic nodules on day 28. **(e)** Immunofluorescence visualization of CD31, αSMA, and NG2 expression in KP tumours from WT and EC-p53KO mice 28 days after fractionated irradiation. Quantification of $\alpha SMA^{+}CD31^{+}$ in total $CD31^{+}$ area (magnification, 200×; n > 5). Scale bar = 50 μm (crop, 10 μm). **(f)** Immunofluorescence visualization of pimonidazole, Ki67 and CD44v6 expression in KP tumours from WT and EC-p53KO mice 28 days after first fractionated irradiation. Relative density of pimonidazole and quantification of $Ki67^{+}CD44v6^{+}$ cells in hypoxic area (magnification, 200×; n > 5). Scale bar = 50 μm. **(g)** Genetic strategy to delete *Trp53* in VE-cadherin-Cre-ERT2 mice. To activate the Cre-ERT2 recombinase, Cre-ERT2 allele-containing mice were injected intraperitoneally with 100 μL tamoxifen solution (2 mg per injection) daily for three consecutive days. WT and CreERT2 EC-p53KO mice received fractionated (2 Gy × 10) or single-dose irradiation (20 Gy). **(h)** Growth of KP tumours in WT and CreERT2 EC-p53KO mice after fractionated or single-dose irradiation. **(i)** Tumour sizes on day 28. **(j)** Immunofluorescence visualization of CD31, αSMA, and NG2 expression in KP tumours from WT and CreERT2 EC-p53KO mice 28 days after first fractionated irradiation. Quantification of $\alpha SMA^{+}CD31^{+}$ in total $CD31^{+}$ area (magnification, 200×; n > 5). Scale bar = 50 μm. **(k)** Immunofluorescence visualization of pimonidazole, Ki67, and CD44v6 expression in KP tumours from WT and CreERT2 EC-p53KO mice 28 days after fractionated or single-dose irradiation. Relative density of pimonidazole and quantification of $Ki67^{+}CD44v6^{+}$ in hypoxic area (magnification, 200×; n > 5). Scale bar = 50 μm. For **(b)**-**(d)**, **(h)** and **(i)**, error bars indicate mean ± SD (Student's t-test). For **(e)**, **(f)**, **(j)** and **(k)**, error bars indicate mean ± SEM (one-way ANOVA for multiple comparision). For all graphs, *p < 0.05, **p < 0.01, ***p < 0.001, ns = not significant. All data are representative of two independent experiments.

Supplementary Figure 10

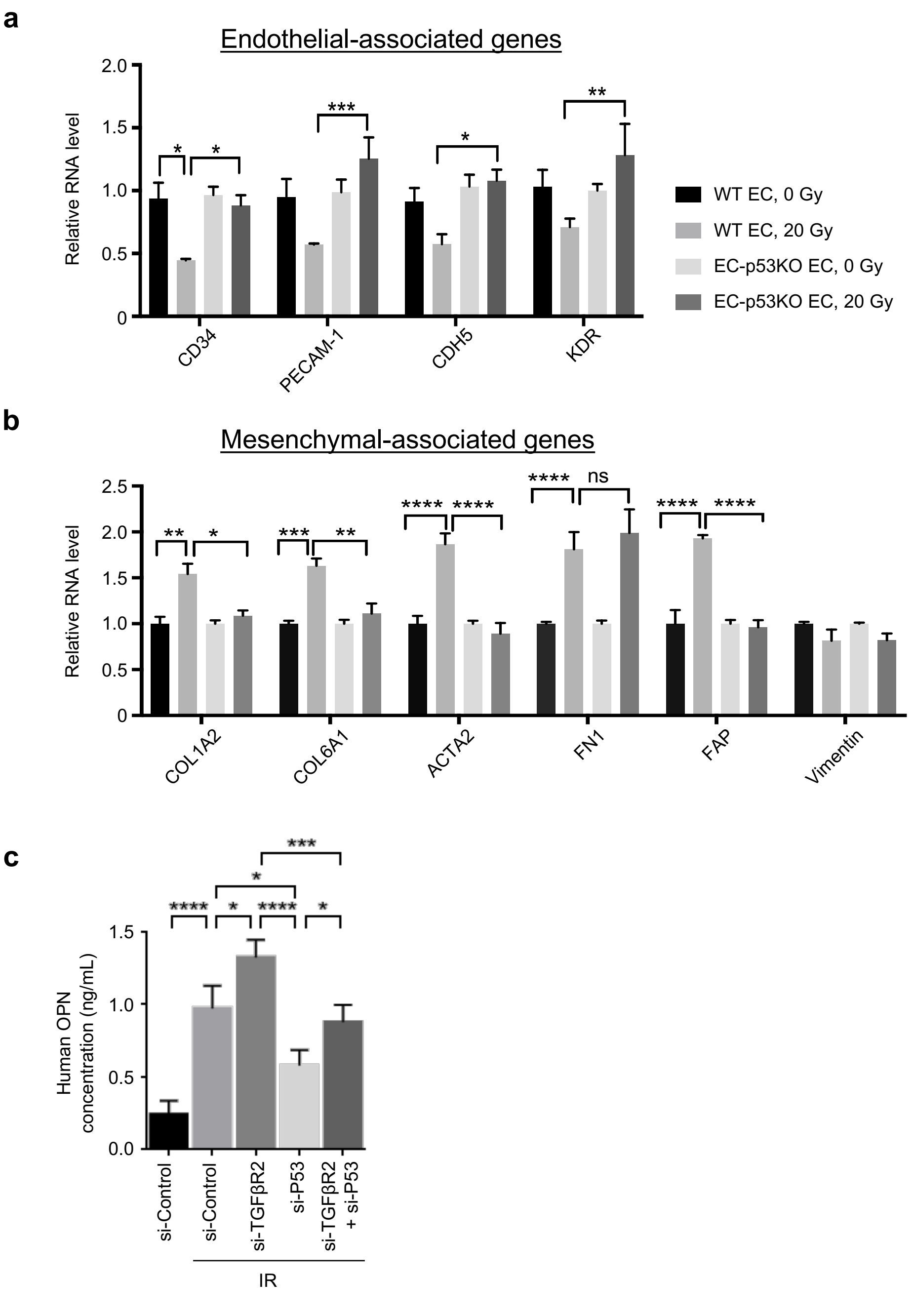

Supplementary Figure 10. Changes in EndMT-related genes in tumour ECs isolated from WT and EC-p53KO mice, and secreted OPN in the supernatants of HUVECs after irradiation. (**a**,**b**) At 8 days after 20 Gy irradiation, total RNA was isolated and subjected to RT-qPCR analysis for the indicated genes. (**c**) At 5 days after 10 Gy irradiation, secreted OPN concentration was measured by ELISA in supernatants from HUVEC-p53KD, TGFβR2KD, or p53+TGFβR2 KD cells. For all graphs, *$p < 0.05$, **$p < 0.01$, ***$p < 0.001$, ****$p < 0.0001$, ns = not significant (one-way ANOVA for multiple comparision). Unless indicated otherwise, error bars indicate mean ± SD. All Data are the means of three independent experiments.

Supplementary Figure 11

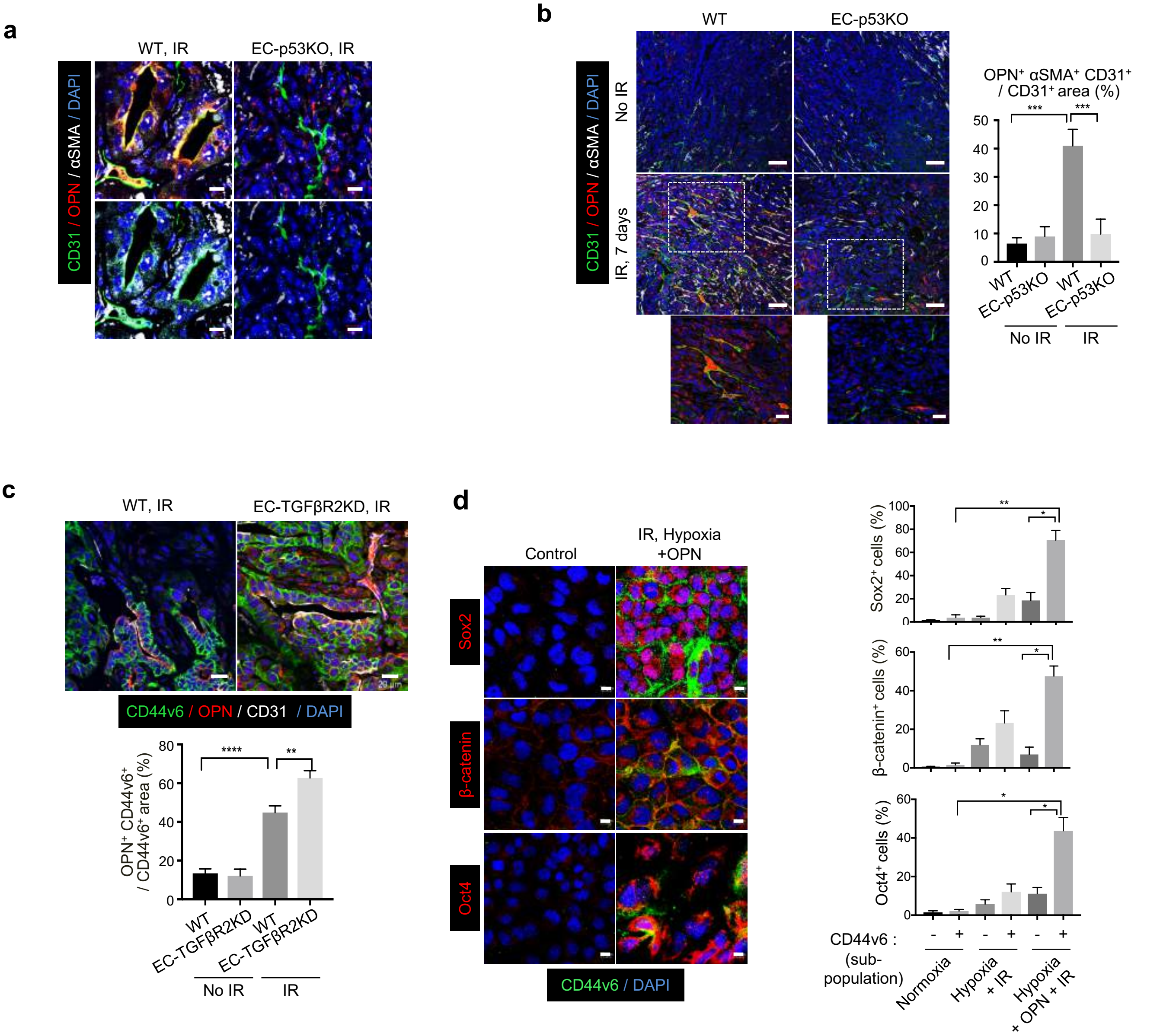

Supplementary Figure 11. OPN expressed during radiation-induced EndMT correlates with increased stemness of CD44v6$^+$ cancer cells **(a)** Immunofluorescence detection of CD31, OPN, and αSMA in KP tumours from irradiated WT and EC-p53KO mice 23 days after 20 Gy irradiation given in a single fraction. Lower panels are CD31 and αSMA images of upper panels. Scale bar = 10 µm. **(b)** Immunofluorescence detection of CD31, OPN, and αSMA in KP tumours from irradiated WT and EC-TGFβR2KD mice (7 days after irradiation). Quantification of OPN$^+$αSMA$^+$CD31$^+$ area in total CD31$^+$ area (magnification, 200×; n > 5). Scale bar = 50 µm (crop, 20 µm). **(c)** Immunofluorescence detection of CD44v6, OPN, and CD31 in KP tumours from irradiated WT and EC-TGFβR2KD mice (24 days after irradiation). Quantification of OPN$^+$CD44v6$^+$ area in total CD44v6$^+$ area (magnification, 200×; n > 5). Scale bar = 20 µm. **(d)** KP tumour cells were cultured under normoxia (20% O_2) or hypoxia (1% O_2) and irradiated with or without OPN. Immunofluorescence detection of stemness proteins (Sox2, β-catenin, and Oct4) and CD44v6 in KP tumour cells 11 days after irradiation. Scale bar = 10 µm. Quantification of Sox2$^+$, β-catenin$^+$ and Oct4$^+$ cells in CD44v6$^+$ or CD44v6$^-$ cells (magnification, 200×; n > 5). For all graphs, *p < 0.05, **p < 0.01, ***p < 0.001, ****p < 0.0001 (one-way ANOVA for multiple comparision). Unless indicated otherwise, error bars indicate the mean ± SEM of three independent experiments.

Supplementary Figure 12

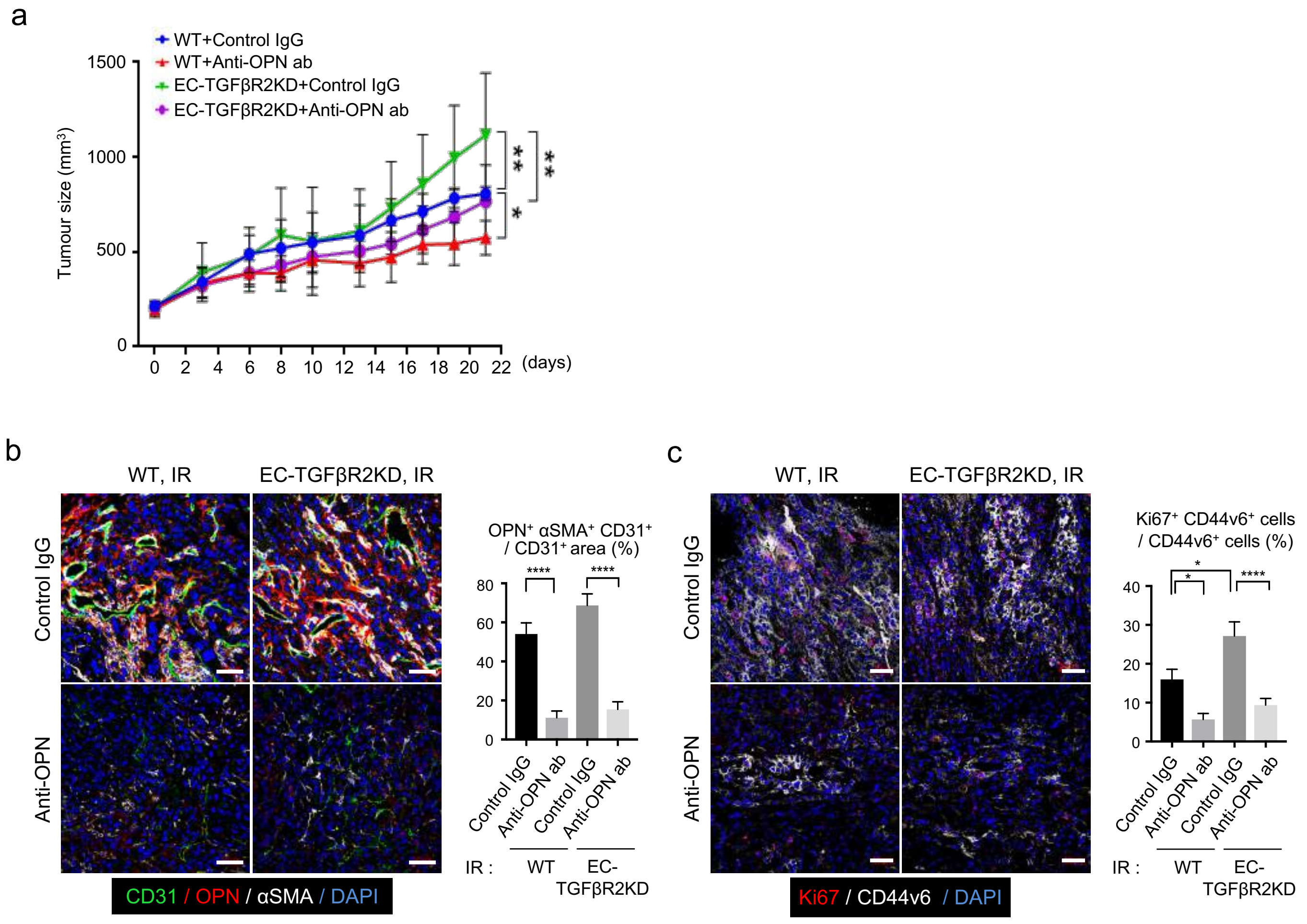

Supplementary Figure 12. Anti-OPN neutralizing antibody attenuates EndMT-mediated CD44v6$^+$ cancer cell proliferation. **(a-d)** KP lung tumour cells were injected subcutaneously into the right hind legs of WT and EC-TGFβR2KD mice. Tumour tissues were obtained 21 days after 20 Gy irradiation given in a single fraction. **(a)** Growth of KP tumours following irradiation. **(b)** Immunofluorescence detection of CD31, OPN, and αSMA in KP tumours from WT and EC-TGFβR2KD mice 21 dpi with or without anti-OPN treatment. Quantification of OPN$^+$αSMA$^+$CD31$^+$ in total CD31$^+$ area (magnification, 200×; n > 5). Scale bar = 50 μm. **(c)** Immunofluorescence detection of Ki67 and CD44v6 in KP tumours from WT and EC-TGFβR2KD mice 21 dpi with or without anti-OPN treatment. Quantification of Ki67$^+$CD44v6$^+$ in total CD44v6$^+$ area (magnification, 200×; n > 5). Scale bar = 50 μm. For **(a)**, error bars indicate mean ± SD. For **(b)** and **(c)** error bars indicate mean ± SEM. For all graphs, *$p < 0.05$, **$p < 0.01$, ****$p < 0.0001$ (one-way ANOVA for multiple comparision). All data are representative of two independent experiments.

Supplementary Figure 13

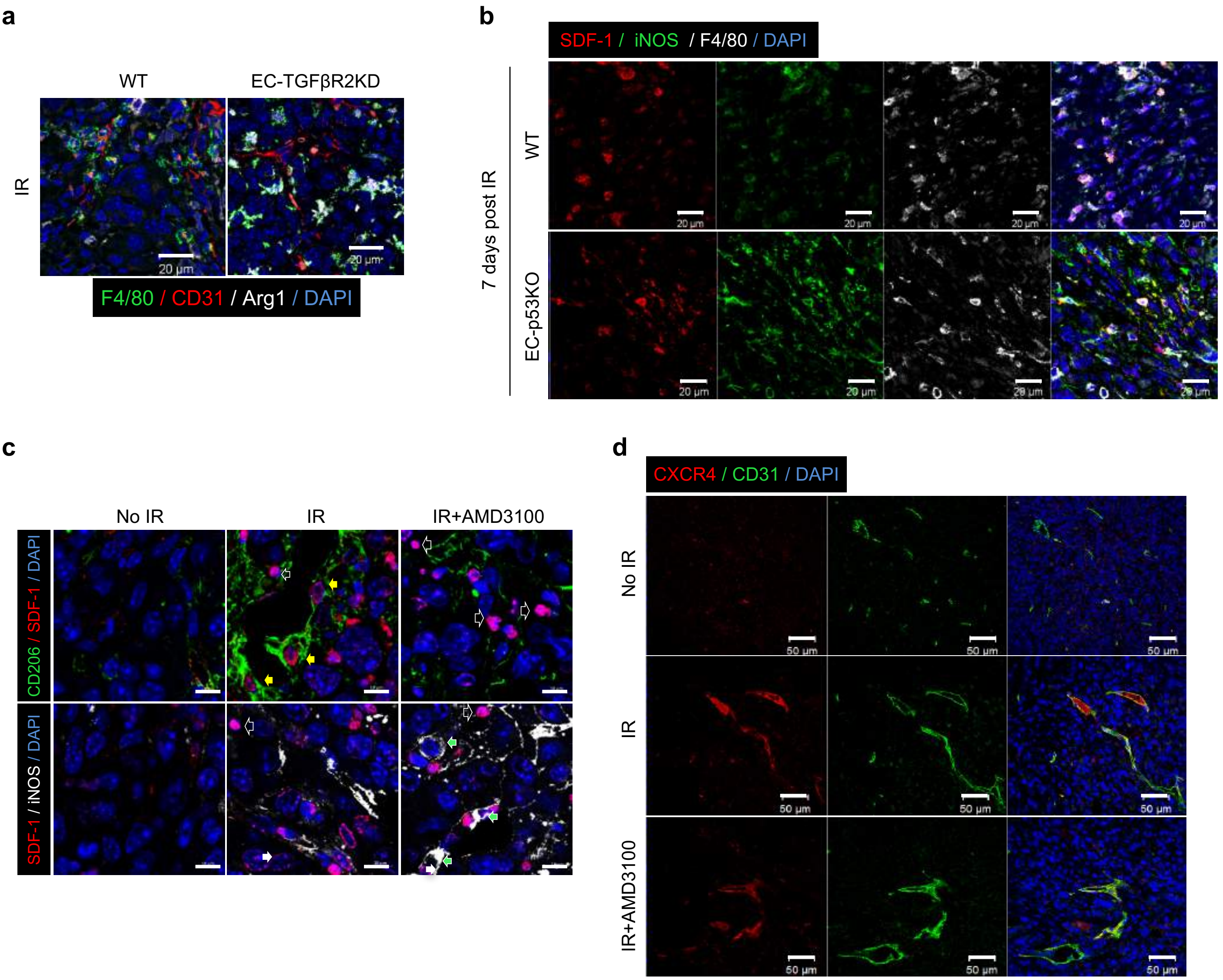

Supplementary Figure 13. M1/M2 populations of TAMs in EC-p53KO and EC-TGFβR2KD tumours and effects of a CXCR4 antagonist on SDF1^{+} M2-subtype cells after 20 Gy irradiation therapy given in a single fraction. (**a**) Immunofluorescence detection of arginase 1 (Arg1), F4/80, and CD31 in KP tumours from WT and EC-TGFβR2KD mice with irradiation (23 dpi). Scale bar = 20 μm. (**b**) Immunofluorescence detection of SDF-1, F4/80, and iNOS in KP tumours from WT and EC-p53KO mice, with or without irradiation (7 dpi). Scale bar = 20 μm. (**c**) Immunofluorescence detection of SDF-1, F4/80, and CD206/CD31 in KP tumours from WT mice 7 dpi, with or without AMD3100 treatment. Scale bar = 10 μm. (**d**) Immunofluorescence detection of CXCR4 and CD31 in KP tumours from WT mice 7 dpi, with or without AMD3100 treatment. Scale bar = 50 μm. All data are representative of three independent experiments

Supplementary Figure 14

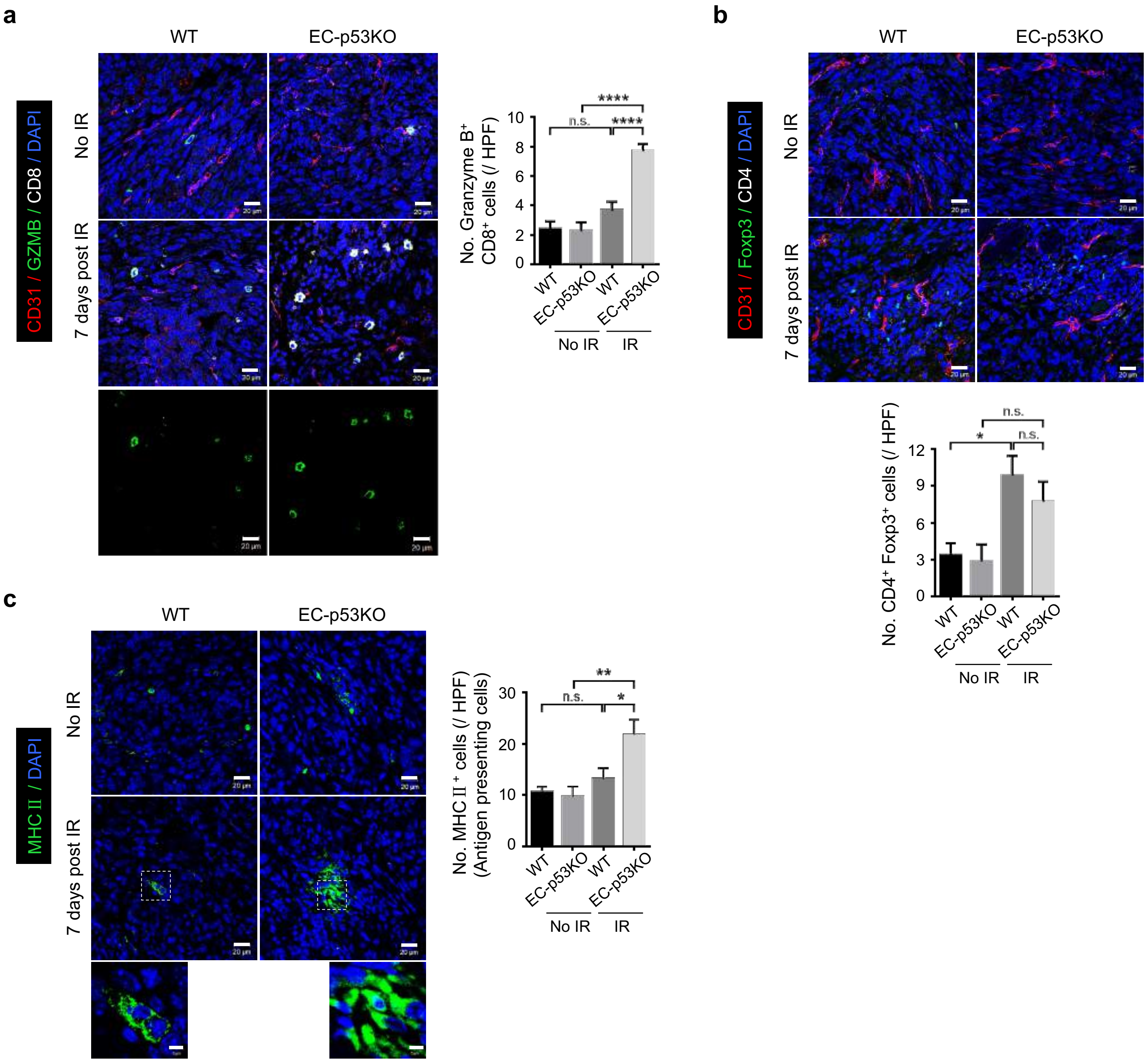

Supplementary Figure 14. Changes in immune cell populations other than macrophages after 20 Gy irradiation given in a single fraction**.** (**a**) Quantification of Granzyme B (GZMB)$^{+}$ CD8^{+} cells (cytotoxic T cells) per field (magnification, 400×; n ≥ 5) on the basis of immunofluorescence images of KP tumours from WT and EC-p53KO mice, with or without irradiation (7 dpi). Scale bar = 20 µm. (**b**) Quantification of CD4^{+} Foxp3^{+} cells (Treg) per field (magnification, 400×; n ≥ 5) using immunofluorescence images of KP tumours from WT and EC-p53KO mice, with or without irradiation (7 dpi). Scale bar = 20 µm. (**c**) Quantification of MHC Ⅱ$^{+}$ cells (APCs) per field (magnification, 200×; n ≥ 5) using immunofluorescence images of KP tumours from WT and EC-p53KO mice, with or without irradiation (7 dpi). Scale bar = 20 µm. Images are representative of 3 independent experiments. IR, irradiated. Tumours were treated with 20 Gy irradiation. For all graphs, *p < 0.05, **p < 0.01, ****p < 0.0001, ns = not significant (one-way ANOVA for multiple comparision). Error bars indicate the mean ± SEM. All data are representative of three independent experiments.

Supplementary Figure 15

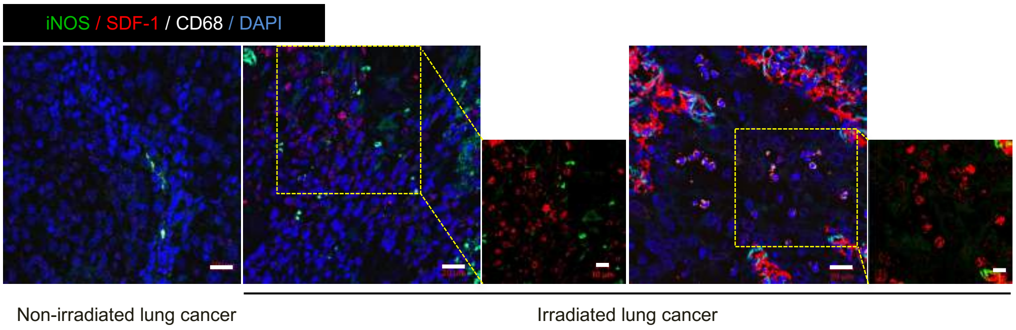

Supplementary Figure 15. M1 polarization of SDF-1^{-} macrophages in human lung cancer tissues from patients treated with or without radiotherapy. Immunofluorescence detection of CD68, SDF-1, and iNOS in irradiated human lung cancer tissues (magnification, 100×). Scale bar = 20 µm (crop, 10 µm). All data are representative of three independent experiments

Supplementary Figure 16

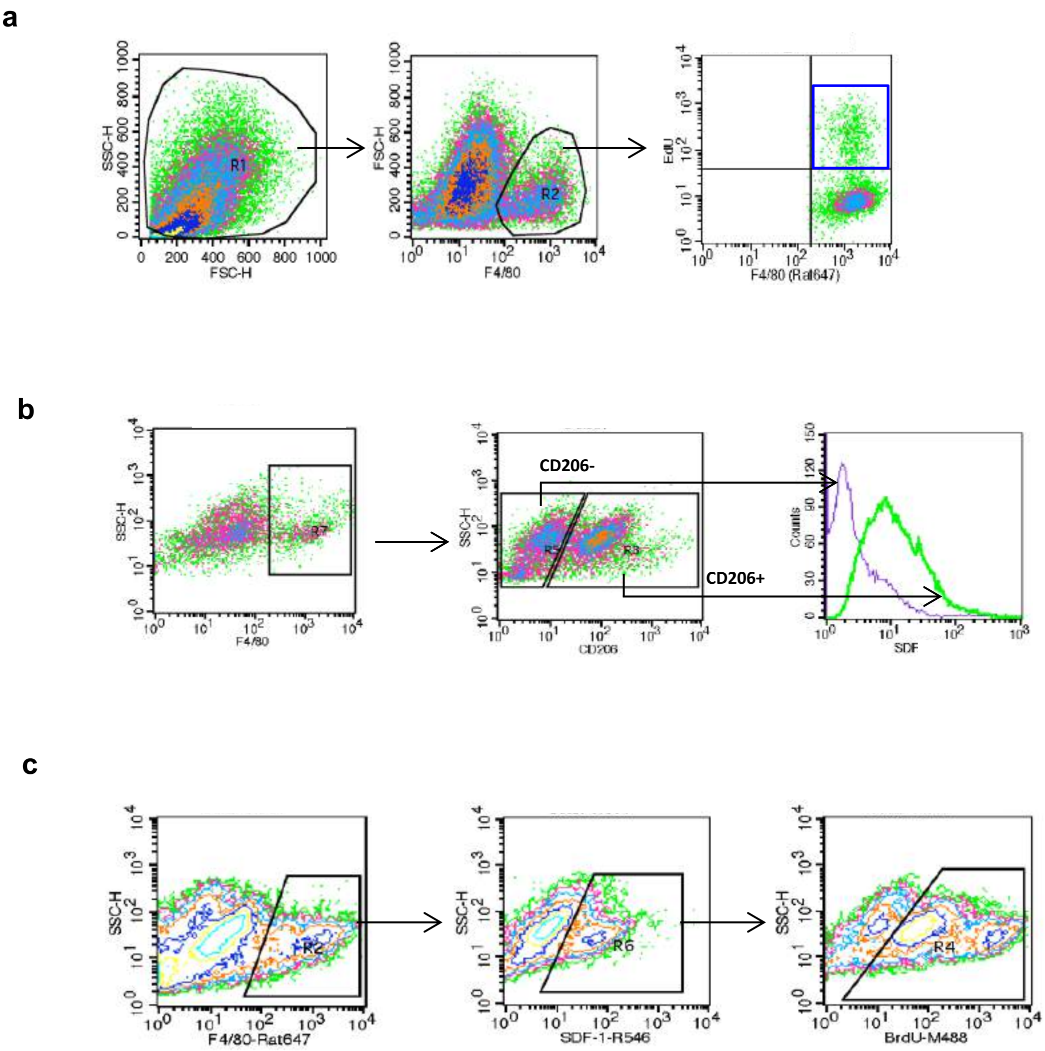

Supplementary Figure 16. Sequential gating strategies for flow cytometry analysis. (**a**) Proliferative ratio of macrophages were identified as F4/80$^+$EdU$^+$ cells in Fig. 5f. (**b**) M2-subtype cells were identifiedas F4/80$^+$CD206$^+$SDF-1$^+$ cells in Fig. 6f. (**c**) Proliferative SDF-1$^+$ macrophages were identified as F4/80$^+$SDF-1$^+$BrdU$^+$ cells in Fig. 6h.

Supplementary Figure 17

Supplementary Figure 1c

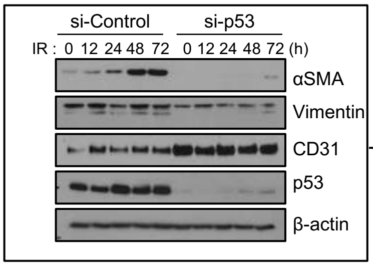

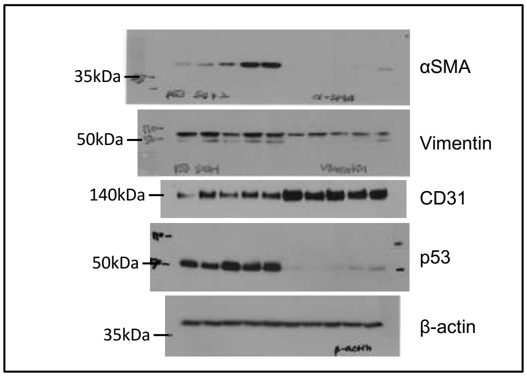

Supplementary Figure 1d

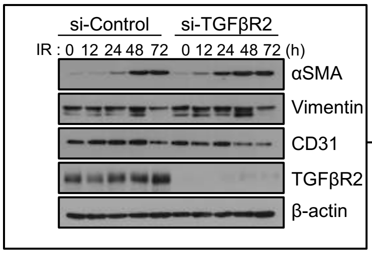

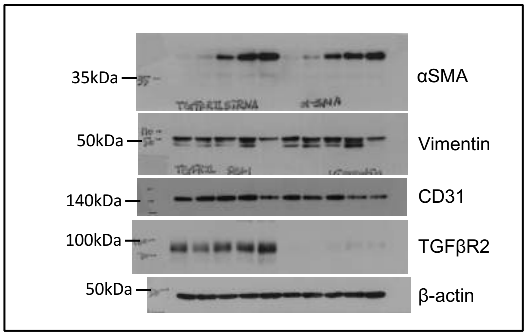

Supplementary Figure 5a

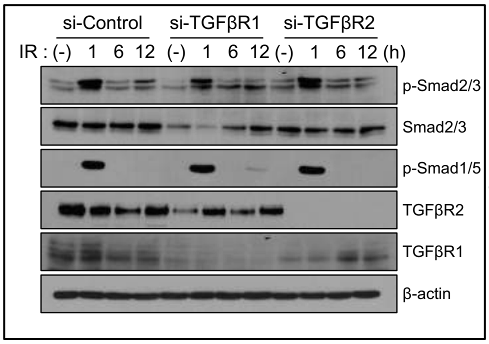

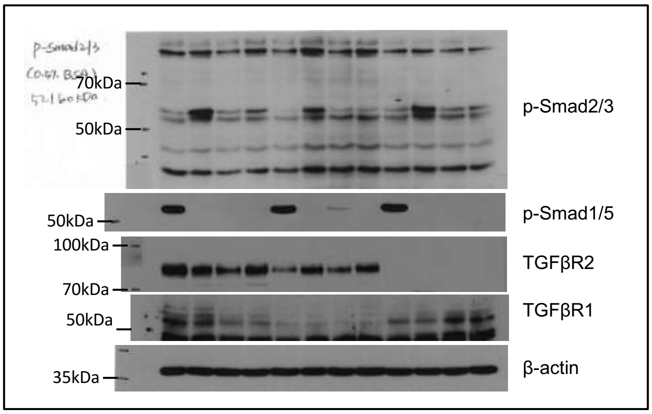

Supplementary Figure 5b

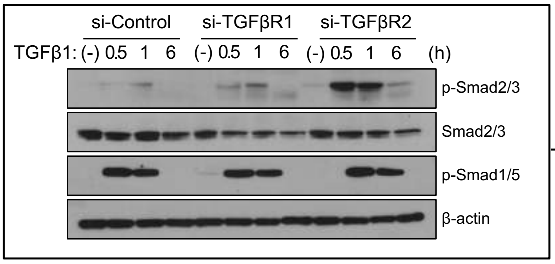

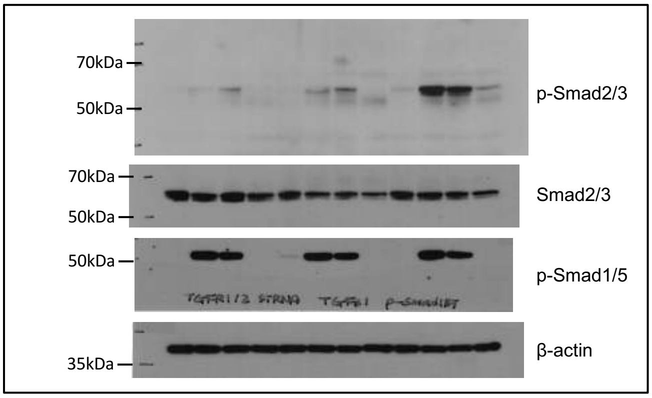

Supplementary Figure 17. Scanned images of key western blots.

Supplementary tables

Supplementary Table 1. Clinicopathologic characteristics of lung cancer patients from whom lung tissues were obtained for analysis in this study

Patient No.	Pathology	Radiation history	Radiotherapy dose	Radiotherapy period	Radiotherapy to surgery interval	Concurrent chemotherapy	Stage	Gender	Smoking history	Oncogenotype
Severance										
# 1	Adenocarcinoma	Yes	50 Gy in 25 fractions	38 days	61 days	Taxotere /Cisplatin	pT2N0M0	F	Non-smoker	EGFR (Exon 21, L858A mutant), KRAS (WT), EML4-ALK translocation (Negative)
# 2	Adenocarcinoma	Yes	54 Gy in 27 fractions	43 days	52 days	Taxotere /Cisplatin	pT1N0M0	M	27 PYR	EGFR (WT), KRAS (WT), EML4-ALK translocation (Negative)
# 3	Squamous cell carcinoma	Yes	45 Gy in 25 fractions	45 days	49 days	Taxotere /Cisplatin	pT4N0M0	M	30 PYR	No information
# 4	Squamous cell carcinoma	Yes	50.4 Gy in 28 fractions	41 days	42 days	Taxotere /Cisplatin	pT1N0M0	M	15 PYR	No information
# 5	Adenocarcinoma	Yes	50 Gy in 25 fractions	36 days	53 days	Paclitaxel /Carboplatin	pT3N1M0	F	Non-smoker	EGFR (E19 del mutant), KRAS(WT), EML4-ALK translocation (Negative)
# 6	Squamous cell carcinoma	Yes	54 Gy in 30 fractions	45 days	104 days	Paclitaxel /Carboplatin	pT1N0M0	M	50 PYR	No information
# 7	Adenocarcinoma	Yes	54 Gy in 27 fractions	41 days	43 days	Taxotere /Cisplatin	pT2N0M0	M	30 PYR	No information
Origene										
# 8	Squamous cell carcinoma	Yes	45 Gy in 28 fractions	28 days		Carboplatin /Navelbine	pT1N1MX	M	30 PYR	No information
# 9	Adenocarcinoma	Yes	45 Gy in 28 fractions	28 days	58 days	Carboplatin /Navelbine,	pT2N2MX	F	30 PYR	No information
# 10	Adenocarcinoma	Yes		Not specified		Carboplatin /Taxol	pT4NXMX	F	30 PYR	No information
# 11	Squamous cell carcinoma	No					pT3N1MX	M	87.5 PYR	No information
# 12	Squamous cell carcinoma	No					pT2N0MX	F	25 PYR	EGFR (Mutant)
# 13	Adenocarcinoma	No					pTXN1MX	M	Smoker: no detailed information	No information
# 14	Adenocarcinoma	No					pT2N1MX	M	53 PYR	No information
# 15	Squamous cell carcinoma	No					pT2N1MX	M	30 PYR	No information
# 16	Squamous cell carcinoma	No					pT2N0MX	M	55 PYR	No information
# 17	Adenocarcinoma	No					pT2N0MX	F	20 PYR	No information
# 18	Squamous cell carcinoma	No					pT2N1MX	M	30 PYR	No information
# 19	Adenocarcinoma	No					pT2N1MX	F	28 PYR	No information
# 20	Adenocarcinoma	No					pT4N0MX	F	52.5 PYR	No information

Notes: p = pathologic TNM stage. PYR = pack-years

Supplementary Table 2. Comparison of clinicopathologic characteristics between patients receiving and patients not receiving radiotherapy

Features	Radiotherapy	No radiotherapy
Number of patients (n)	10	10
Sex		
Male (%)	6 (60.0)	6 (60.0)
Female (%)	4 (40.0)	4 (40.0)
Carcinoma type		
Adenocarcinoma	6 (60.0)	5 (50.0)
Squamous cell carcinoma	4 (40.0)	5 (50.0)
Tumour stage		
T0 (%)	0 (0.0)	0 (0.0)
T1 (%)	4 (40.0)	0 (0.0)
T2 (%)	3 (30.0)	7 (70.0)
T3 (%)	1 (10.0)	1 (10.0)
T4 (%)	2 (20.0)	1 (10.0)
Tx (%)	0 (0.0)	1 (10.0)
Nodal status		
N0 (%)	6 (60.0)	4 (40.0)
N1 (%)	2 (20.0)	6 (60.0)
N2 (%)	1 (10.0)	0 (0)
Nx (%)	1 (10.0)	0 (0)
Smoking history		
Non-smoker (%)	2 (20.0)	1 (10.0)
Smoker (%)	8 (80.0)	9 (90.0)
PYR of smoker, median (min–max)	30 (15–30)	30 (20–87.5)

Supplementary Table 3. Sequences of primers used for RT-qPCR

A. **Human genes**

Gene	Accession No.	Forward primer (5'–3')	Reverse primer (5'–3')
PECAM1	NM_000442.4	CCCAGGAGTTTCCAGAAATC	TTGTTGCCATGTCTGTTGTG
CDH5	NM_001795.4	CAGTTTGACCGGGAGCATAC	GACTTGGCATCCCATTGTCT
TEK	NM_000459.4	CCCTCCTCCAAGAGGTCTAA	ATATTGGTTGCCAGGTCAAA
VWF	NM_000552.4	TGCCTCAGGAAAGAAAGTCA	CAACATCACAGTGGCAAATC
CD34	NM_001025109.1	TAGCCTGTCACCTGGAAATG	GGGTTTAGTGGGAGATGTTG

COL1A2	NM_000089.3	CATGGGACCCAGAGGTCTTC	GCCGACAGGACCTTCTTTTC
COL5A1	NM_000093.4	CGTGGGAAACTGCTCTCCTA	CCGCAGGAAGGTCATCTGTA
COL5A2	NM_000393.4	AGCACTGGTCCTCAGGGAAT	GCCAGCTTCTCCTTTGAAAC
COL6A1	NM_001848.2	CGTCGATGCCATGGACTTTA	CGGTAGAAGCGGGTCACATA
FN1	NM_001306131.1	TGAGGCAACGTGTTATGATG	GCACCGAGATATTCCTTCTG
VIM	NM_003380	GAACGCCAGATGCGTGAAAT	GCGGCCAATAGTGTCTTGGT
FAP	NM_004460	GATGGACGCACTGATCAAGA	GGCAGCTGGATATTTTTCAA
SNAI1	NM_005985	CAAGGATCTCCAGGCTCGAA	GGCACCCAGGCTGAGGTATT
SNAI2	NM_003068	GCAAAAACTGCTCCAAAACC	TACACAGCAGCCAGATTCCT
ZEB1	NM_001174096	CTGCCAACAGACCAGACAGT	TCCCAGCAGTTCTTAGCATT
ZEB2	NM_001171653	TTCAGGGAGAATTGCTTGAT	CAGGAGTCGGAGTCTGTCAT
ACTA2	NM_001141945.2	CTGCTGAGCGTGAGATTGTC	TGGCCATCTCATTTTCAAAG

B. Mouse genes

**Gene**	**Accession No.**	**Forward primer (5'–3')**	**Reverse primer (5'–3')**
Pecam1	NM_008816	GGGCACACCTGTAGCCAACT	CTCGGCGATCTTGCTGAAAT
Cdh5	NM_009868.4	GAACGAGGACAGCAACTTCA	CCCATACTTGACCGTGATGT
Kdr	NM_010612.2	GACCCCAAATTCCATTATGA	GGCTCTTTCGCTTACTGTTC
Cd34	NM_001025109.1	TAGCCTGTCACCTGGAAATG	GGGTTTAGTGGGAGATGTTG
Col1a2	NM_007743.3	TGGAATCCGAGGTCCTAATG	AAGACCTCTGGGTCCCATGA
Col6a1	NM_009933.4	TTCATCAACGACGCCACAGA	ACGAGGCTTCCCGGTAGAAA
Fn1	NM_001276410.1	GTGCTATGACGATGGGAAGA	GCAAATGGCTCCGAGATATT
Vim	NM_011701.4	TCAAACGAGTACCGGAGACA	GGGACTCGTTAGTGCCTTTA
Fap	NM_007986.3	AAGGAAAGAAAGGTGCCAAT	CCATAGCAGACGAGTGCATA
Acta2	NM_007392.3	CTGACTGAGCGTGGCTATTC	GCACAGCTTCTCCTTGATGT
